# Surveillance of Human Echinococcosis in Castilla-Leon (Spain) between 2000-2012

**DOI:** 10.1371/journal.pntd.0004154

**Published:** 2015-10-20

**Authors:** Amparo Lopez-Bernus, Moncef Belhassen-García, Montserrat Alonso-Sardón, Adela Carpio-Perez, Virginia Velasco-Tirado, Ángela Romero-Alegria, Antonio Muro, Miguel Cordero-Sánchez, Javier Pardo-Lledias

**Affiliations:** 1 Servicio de Medicina Interna, CAUSA, CIETUS, IBSAL, Salamanca. Spain; 2 Servicio de Medicina Interna, Sección de Enfermedades Infecciosas, CAUSA, CIETUS, IBSAL, Salamanca, Spain; 3 Área de Medicina Preventiva y Salud Publica, CIETUS, IBSAL, Universidad de Salamanca, Salamanca, Spain; 4 Laboratorio de Inmunología Parasitaria y Molecular, CIETUS, IBSAL, Facultad de Farmacia, Universidad de Salamanca, Salamanca, Spain; 5 Servicio de Medicina Interna, CAUPA Hospital General de Palencia “Río Carrión,” Palencia, Spain; University of Zurich, SWITZERLAND

## Abstract

**Background:**

Cystic echinococcosis (CE) is an important health problem in many areas of the world including the Mediterranean region. However, the real CE epidemiological situation is not well established. In fact, it is possible that CE is a re-emerging disease due to the weakness of current control programs.

**Methodology:**

We performed a retrospective observational study of inpatients diagnosed with CE from January 2000 to December 2012 in the Western Spain Public Health-Care System.

**Principal findings:**

During the study period, 5510 cases of CE were diagnosed and 3161 (57.4%) of the cases were males. The age mean and standard deviation were 67.8 ± 16.98 years old, respectively, and 634 patients (11.5%) were younger than 45 years old. A total of 1568 patients (28.5%) had CE as the primary diagnosis, and it was most frequently described in patients <45 years old. Futhermore, a secondary diagnosis of CE was usually found in patients >70 year old associated with other causes of comorbidity. The period incidence rate was 17 cases per 10^5^ person-years and was significantly higher when compared to the incidence declared through the *Notifiable Disease System* (1.88 cases per 10^5^ person-years; p<0.001).

**Conclusions:**

CE in western Spain is an underestimated parasitic disease. It has an active transmission, with an occurrence in pediatric cases, but has decreased in the recent years. The systematic search of Hospital Discharge Records of the National Health System Register (HDR) may be a more accurate method than other methods for the estimation of the incidence of CE in endemic areas.

## Author Summary

The incidence of CE in our region is still high; however, in this period of study, a slow decrease was observed. The sharp decline of incidence in pediatric population highlights the importance of long-term control of CE. The systematic search of HDR may be a more accurate method than other methods in the estimation of the incidence of CE in endemic areas.

## Introduction

Human echinococcosis is a zoonotic infection caused by cestodes of the genus *Echinococcus* sp. Four species infect humans: cystic echinococcosis (CE) is caused by *Echinococcus granulosus*, alveolar echinococcosis (AE) is caused by *E*. *multilocularis*, and polycystic forms are caused by either *E*. *vogeli* or *E*. *oligarthrus*; however, they are less frequently associated with human infection.

CE is considered a neglected disease whose clinical manifestations range from asymptomatic infection to severe disease [1–3]. Although CE is considered an eradicable parasite, CE remains a considerable health problem in endemic regions with substantial economic losses for agricultural sectors and public health systems [4]. CE occurs worldwide; however, this disorder is endemic in central Asia, northern and eastern Africa, Australia, South America and the Mediterranean basin [5–7]. The transmission rate of *E*. *granulosus* in Spain remains high, and it is considered a highly endemic area inside the European region [8]. The central, northeastern and western regions of Spain are the most important endemic regions, such as Castilla-Leon, where extensive or semi-extensive farming of livestock (mostly sheep) is common [8,9]. Since the mid-1980s, several prevention and control campaigns have been implemented to reduce *E*. *granulosus* infection in Spain[10]. The epidemiological methods used in the evaluation of human hydatidosis were based, mainly, on notifiable cases system and detection of cases from Hospital Discharge Records (HDR) [9,10]. In a recent study, in a province of western Spain, we studied the evolution of incidence during fifteen years using both methods, and we detected an important under-notification of CE cases [11]. Thus, the aim of this study was to compare these epidemiological methods for evaluation of CE in a region of western Spain and to determine the evolution of the incidence over thirteen years.

## Materials and Methods

The design was a retrospective observational study of inpatients diagnosed with CE in the Western Spain Public Health-Care System from January 2000 to December 2012.

This study was conducted in Castilla-Leon, a region placed in western Spain, between parallels 40° 05' and 43° 14' North latitude, and 1° 46' and 7° 05' West, which covers an area of 94.223 square kilometers and has a population of 2,558,463 persons. Of the people in this area, 1,661,817 live in municipalities with more than 5,000 inhabitants (defined as an urban population) and 896,646 live in municipalities with less than 5,000 inhabitants (defined as a rural population) (INE; Census, January 1, 2011 http://www.ine.es). The public health service covers 2,455,323 inhabitants (95.96% of this population) and includes 118,383 (4.82%) foreigners (http://www.saludcastillayleon.es/). Regarding territorial organization, this area consists of 9 provinces with 2,248 municipalities and 59 municipalities with a population of over 5,000 habitants (Junta de Castilla-Leon; http://www.jcyl.es). The public specialized health-care system includes 14 hospitals.

The data were collected from HDR. Patients with missing data, such as age, gender or the city of residence, and residents from other regions of Spain were excluded from the study. For a better analysis, patients were stratified according to gender and age (0–19, 20–44, 45–69, and ≥70 years). Urban origin was defined when place of residence had >5000 inhabitans. The data were analyzed anonymously.

This study analyzes and compares the data provided by two existing surveillance systems in Spain: HDR and Notifiable Disease System (NDS). In Spain, the surveillance of communicable/transmissible diseases is regulated by the National Epidemiological Surveillance Network (National Center for Epidemiology, Carlos III Institute of Health, Ministry of Health, Social Services and Equality). Data are received, elaborated and transmitted to European Centre for Disease Prevention and Control (ECDC) there.

The HDRs provides information about patients who have been admitted to the hospital during the years 2000–2012. We have collected information of hospital admissions with EC code (ICD-9: 122.0–122.9) in diagnostics report of discharge. When patients have been admitted with the same diagnosis several times, we used to register only the first admission for analysis. Hydatid disease is a disease of compulsory numerical declaration so that the Notifiable Disease System (NDS) has only the number of cases per week and per year, which allows to calculate incidence rates per 100,000 population.

### Statistical analysis

The annual/period incidence rate of CE was calculated by dividing the number of new cases of disease observed in the defined time period (1 year or 13 years, respectively) by the total free periods of disease-person time during the observation period defined in the study, multiplied by 100,000 and expressed as “cases per 10^5^ person-years”. As it is not possible to accurately measure disease free periods, the total figure of person-time at risk can be estimated approximately and satisfactory when the size of the population is stable, multiplying the average population size studied by the duration of the observation period. Thus, the denominators were obtained from population counts for each year at the municipality level of the National Institute of Statistics (INE; http://www.ine.es/).

The results were expressed as percentages (with corresponding 95% confidence interval, 95% CI, for a proportion) for categorical variables and as the mean and standard deviation (SD) for continuous variables. A chi-square test was used to compare the association between categorical variables, such as clinical and demographics variables, and the measured outcome was expressed as the odds ratio (OR) together with the 95% CI for OR. Continuous variables were compared with Student’s t-test or the Mann-Whitney for two groups, depending on their normal or non-normal distribution. Additionally, we applied the corresponding regression models for multivariate analysis. We considered a statistically significant difference from chance at a p-value <0.05. All of the data were analyzed with SPSS 21 (*Statistical Package for the Social Sciences*).

### Ethics statement

This study was approved by the Ethics Committee of Complejo Asistencial Universitario de Salamanca (CAUSA). Due to it is an epidemiological study, the written consent was not obtained and it was specifically waived by the approving IRB. All data analyzed were anonymized.

## Results

Between January 2000 and December 2012, 5510 patients with CE were registered with HDR in the 14 hospitals. The main demographic data of the participants are shown in **Table 1**.

**Table 1:** Principal demographic data in patients included in the study.

Demographic data	All patients N = 5510
Male sex	3161 (57.4%)
Mean Age ± SD	67.8 ± 16.98
0–19 years	51 (0.9%)
20–44 years	583 (10.6%)
45–69 years	1791 (32.5%)
>70 years	3085 (56.0%)
Patients from rural areas (<5000 inhabitans)	2873 (52.1%)

**Fig 1:**
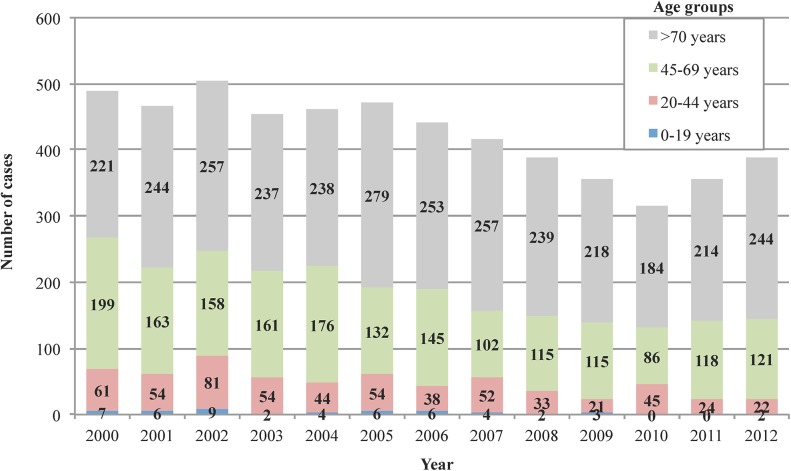
Annual cases of CE in Castilla-Leon (western Spain) according to the age and year distribution.

Fifty-one diagnosed patients (0.9%) were children or adolescents (0–19 years), 583 patients (10.6%) were between 20–44 years old, 1791 patients (32.5%) were between 45–69 years old and 3085 patients (56.0%) were ≥70 years old (**Fig 1)**. Collectively, the young had a higher probability of being male OR = 1.2 (95% CI, 1.055–1.483; p = 0.010).

**Fig 2:**
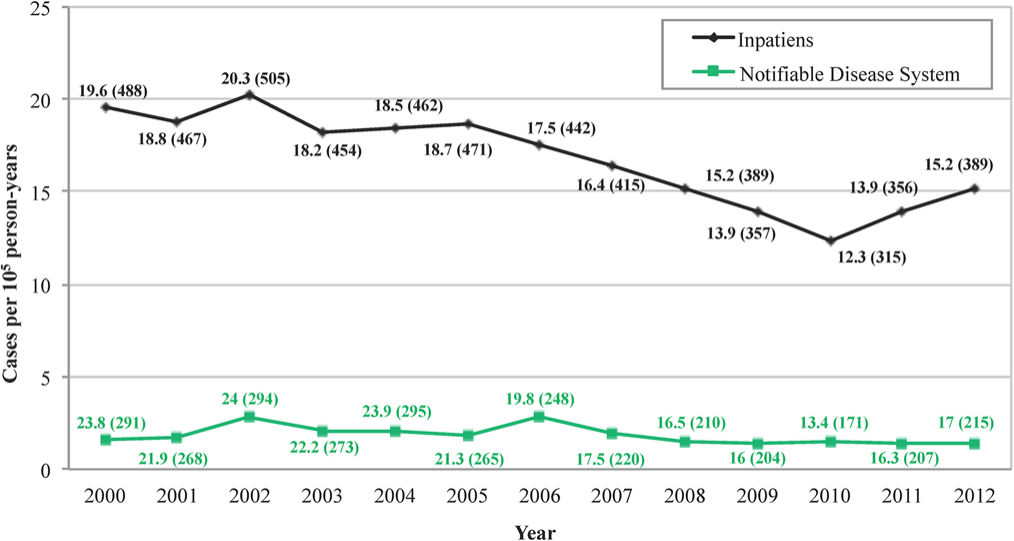
Cases per 10^5^ person-years in Castilla-Leon (western Spain) measured by hospital admissions and “Notifiable Disease System”.

The period incidence rate was 17 cases per 10^5^ person-years (5510 cases), which was significantly higher than the data reported by the *“Notifiable Disease System”* (17 cases per 10^5^ person-years *versus* 1.88 cases per 10^5^ person-years, (p<0.001)) as shown in **Fig 2**.

A progressive decrease in the incidence of CE was detected, from 19.6 cases per 10^5^ person-year in the 2000 to as low as 12.3 cases per 10^5^ person-year in the 2010, although this incidence has increased in the last two years (**Fig 2**). According to these data, a decrease in the diagnosis of new cases in individuals <45 years old was found from 2007–12 (382 *versus* 208 cases; OR = 1.49; 95% CI, 1.254–1.793; p<0.001), with a more pronounced decline in the pediatric population (34 *versus* 11 cases; OR = 2.42; 95% CI, 1.227–4.803; p = 0.008). Regarding the origin areas, 2,873 (52.1%) patients were residents in rural areas, whereas 2,637 (47.9%) cases came from urban areas, and the incidence of CE in rural areas was twice as much as that in urban areas (24.6 cases per 10^5^ person-years *versus* 12.2 cases per 10^5^ person-years, p<0.001). A logistic regression model revealed significant differences in relation to gender (p<0.001) and age (p = 0.003), with more frequent rural origin among men (OR = 1.36; 95%CI, 1.22–1.52) and those individuals older than 70 years (OR = 1.17; 95% CI, 1.05–1.31).

The most frequent location of CE was the liver with 4,364 patients (79.1%). We further classified the patient’s diagnosis of CE according to ICD-9 as shown in **Table 2.**

**Table 2:** Classification of echinococcosis in a region of western Spain over thirteen years.

International Classification of Diseases (ICD-9)	Patients n (%)
122.0 *E*. *granulosus* hepatic infection	1115 (20.2)
122.1 *E*. *granulosus* pulmonary infection	201 (3.7)
122.2 *E*. *granulosus* thyroid infection	19 (0.3)
122.3 *E*. *granulosus* another infection	113 (2.1)
122.4 *E*. *granulosus* unspecified infection	12 (0.2)
122.5 *E*. *multilocularis* hepatic infection	22 (0.4)
122.6 *E*. *multilocularis* another infection	5 (0.1)
122.7 *E*. *multilocularis* unspecified infection	0 (0)
122.8 Unspecified hepatic echinococcosis	3223 (58.5)
122.9 Other unspecified echinococcosis	800 (14.5)
**Total**	5510

CE was the primary diagnosis and the main cause of hospitalization in 1568 (28.5%) patients, and CE was a secondary diagnosis in 3,942 (71.5%) of the cases.

Patients younger than 45 years of age had a more frequent primary diagnosis of CE than did patients older than 45 years o age (72.2% *versus* 22.8%; OR = 8.8; 95% CI, 7.329–10.637, p<0.001).

Ninety-five percent (5231) of the patients had at least one chronic disease. The average number of diseases per patient with CE was 5.87 [interval range: 1–9]. The most common chronic diseases were cancer (1561, 28.3%), heart failure (461, 8.3%), atrial fibrillation (562, 10.2%), cerebrovascular disease (317, 5.7%), chronic obstructive pulmonary disease (704, 12.8%), diabetes mellitus (770, 14%), and chronic kidney failure (180, 3.3%).

## Discussion

CE is a worldwide zoonotic infection that affects human and animal health, and it is the cause of significant economic loss for the agricultural sectors and public health systems in the endemic area [8]. Recent studies have shown that CE is a re-emerging disease in several countries and regions, even in places where the prevalence was previously low[5,6,11].

It has been demonstrated that control campaigns based on health education, control, elimination of the slaughter of sheep at home, a change in risk behaviors, such as elimination of stray dogs, the reduction of parasite biomass in the definitive hosts (by administering praziquantel) and the removal of animal corpses, may decrease the incidence and prevalence of infection by CE [10,12–15]. The reduction of these programs due to the lack of economic resources may have catastrophic consequences, leading to severe disease, considerable economic loss, and a definite public health problem of increasing concern [6]. Thereby, the WHO is working toward the validation of effective cystic echinococcosis control strategies by 2018.

Historically, CE in Spain is one of the most important existing anthropozoonoses, and western Spain is a region with a highly endemic occurrence due to extensive or semi-extensive farming of livestock and the *E*. *granulosus* cycle and its continuation over many years [9]. To the best of our knowledge, the autochthonous transmission in Spain is only by *E*.*granulosus* (never by *E*. *multilocularis* nor other species), therefore, the reported cases of *E*. *multilocularis* is probably due to misclassification or less likely to imported cases originating from an endemic country. Unfortunately, given the characteristics of the study these results can not be assessed.

Our group, using HDR detected a number of local cases that were not previously identified due to a lack of notification [11]. In this work, we also compared these two epidemiological methods in a wide area with almost 2.5 million inhabitants to determine the incidence of CE during 2000 to 2012. Thus, according our previous work, we used HDR and we detect a higher incidence of CE than that detected by the “Notifiable Disease System”. A low percentage of surgical cases detected in other studies (<70%) supports the fact that HDR is at the moment the most accurate method in the evaluation of health campaigns regarding echinococcosis. Methods based on serological or ultrasonographic screening have been used to study the prevalence of CE in different areas [16–18], but these methods are more expensive and cannot be used in large populations over multiple years to establish the epidemiological evolution of echinococcosis in humans.

The initiative European formally named FP7 project HERACLES (Human cystic Echinococcosis Research in Central and Eastern Societies), was born in 2013 [19,20]. One of the most important objectives was create the European Registry of Cystic Echinococcosis (ERCE). However, in this moment, the participation of groups that diagnose and treat to patients is not assured. In this sense, until the results of this registry are published, we think the HDR system may be the best method for surveilling CE in our area.

Thus, in our work, we showed that in the study period, the incidence of CE in this region had a slow reduction. According to these data, we found a decrease in the diagnosis of new cases younger than 45 years old, with a decline of almost half the number of cases between 2007–2012 compared to 2000–2005. This decrease is still higher in the pediatric population with a reduction to one-third the number of cases. These results show that campaigns of public health, based on the elimination of stray dogs and especially the removal of animal corpses (implemented after the crisis of bovine spongiform encephalopathy), may decrease the incidence of infection in a wide endemic area and help control CE [10].

However, our data support that the economic burden of CE in Spain was clearly underestimated; Benner et al. estimated the economic losses due to CE in Spain in 2005 at 148.9 million euros[12,21], and the diagnosed cases of CE were nearly triple in the same period in our region.

Despite the wide distribution of cases in our region, we found a higher cumulative incidence in rural than in urban areas and this pattern of CE infection has also been documented in previous studies [22]. Most patients with CE were living in rural areas with a wide geographic distribution. This heterogeneity on the geographic distribution of CE has also been reported in numerous countries; therefore, it is difficult to identify risk factors for this disease in our province, region and country [23]. Additionally, we detected that the disease incidence is very similar in both sexes, suggesting that the occupational component of the risk is less relevant than other risk factors attributable to environmental conditions [22]. This result supports that health educational strategies must be intensified, especially in rural areas.

Regarding the diagnosis of CE, we found that the primary diagnoses of CE were performed in young patients, while the secondary accidental diagnosis was most frequently found in the elderly population and usually associated with other causes of comorbidity. Despite being traditionally considered as a “benign” pathology, CE is an important cause of morbi-mortality in patients older than 65 years [1]. Thereby, the diagnosis of CE in the elderly population is usually understimated. Therefore, an expectant management of the disease can be dangerous, and it must be only employed in select patients.

Additionally, we detected that the patients that were primarily admitted for CE are approximately third of the cases, with the remainder being a secondary diagnosis with the patient admitted for some other reason. This means that that nearly two third of the CE cases was an incidental finding. This is indicative that a large numbers of patients with echinococcosis who remain undiagnosed, and is a further evidence that the disease is under reported.

The main limitation of our work was the initial selection bias. The present study only considers the cases admitted to public hospital care; cases of private clinics and primary care were not included in this study. Therefore, we can assume that the actual incidence of human hydatidosis is even higher than the incidence estimated in this study.

One aspect to assess is the immigration impact on these results, which can not be unavailable by the HDR. Data from our center show that immigration has limited impact, with figures around 3% (A. Romero-Alegria, M. Belhassen-Garcia, Supporting Information).

It can be concluded that the systematic search of HDR may be a more accurate method than other methods, based on the notification of cases in the estimation of the incidence of CE in endemic areas. The incidence of CE in our region is still high; however, in this period of study, a slow decrease was observed. The sharp decline of incidence in pediatric population highlights the importance of long-term control of CE.

## Supporting Information

S1 Checklist STROBE checklist. (DOC)

S1 File Impact of imported hydatidosis. (DOCX)
