# Supplementary material for: Surveillance of Human Echinococcosis in Castilla-Leon (Spain) between 2000-2012
Source: PLoS Negl Trop Dis. 2015 Oct 20;9(10):e0004154. doi: 10.1371/journal.pntd.0004154 (PMC4618931; doi:10.1371/journal.pntd.0004154)
Supplement: S1 Checklist — (DOC) [file pntd.0004154.s001.doc]

Research checklist. STROBE STATEMENT

Surveillance of Human Echinococcosis in Castilla-Leon (Spain) between 2000-2012.

|  | Item No | Recommendation |
| --- | --- | --- |
| **Title and abstract** | 1 | (*a*) We have indicate the study’s design with a commonly used term in the abstract |
|  |  | (*b*) We have provided in the abstract an informative and balanced summary of what was done and what was found |
| Introduction | | |
| Background/rationale | 2 | We have explained the scientific background and rationale for the investigation being reported |
| Objectives | 3 | We have stated specific objectives |
| Methods | | |
| Study design | 4 | We have presented key elements of study design early in the paper |
| Setting | 5 | We have described the setting, locations, and relevant dates, including periods of recruitment, exposure and data collection |
| Participants | 6 | We have given the eligibility criteria, and the sources and methods of selection of participants |
|  |  |  |
| Variables | 7 | We have clearly defined all outcomes, exposures, predictors, potential confounders, and effect modifiers. |
| Data sources/ measurement | 8 | For each variable of interest, we have given sources of data and details of methods of assessment (measurement). We have described comparability of assessment methods if there is more than one group |
| Bias | 9 | We have described efforts to address potential sources of bias |
| Study size | 10 | We have explained how the study size was arrived at |
| Quantitative variables | 11 | We have explained how quantitative variables were handled in the analyses. |
| Statistical methods | 12 | (*a*) We have described all statistical methods, including those used to control for confounding |
|  |  | (*b*) We have described methods used to examine subgroups and interactions |
|  |  | (*c*) We have explained how missing data were addressed |
|  |  |  |
|  |  | (d) We have described any sensitivity analyses |

Continued on next page

| Results | | |
| --- | --- | --- |
| Participants | 13 | (a) We have reported numbers of individuals at each stage of study |
|  |  |  |
|  |  |  |
| Descriptive data | 14 | (a) We have given characteristics of study participants (eg demographic, clinical, social) and information on exposures and potential confounders |
|  |  | (b) We have indicated number of participants with missing data for each variable of interest |
|  |  |  |
| Outcome data | 15 |  |
|  |  |  |
|  |  |  |
| Main results | 16 | (*a*) We have given unadjusted estimates and confounder-adjusted estimates and their precision (eg, 95% confidence interval). |
|  |  | (*b*) We have reported category boundaries when continuous variables were categorized |
|  |  |  |
| Other analyses | 17 | We have reported other analyses done |
| Discussion | | |
| Key results | 18 | We have summarised key results with reference to study objectives |
| Limitations | 19 | We have discussed limitations of the study, taking into account sources of potential bias or imprecision. |
| Interpretation | 20 | We have given a cautious overall interpretation of results considering objectives, limitations, multiplicity of analyses, results from similar studies, and other relevant evidence |
| Generalisability | 21 | We have discussed the generalisability (external validity) of the study results |
| Other information | | |
| Funding | 22 | Not applicable |
