# Supplementary material for: Surveillance of Human Echinococcosis in Castilla-Leon (Spain) between 2000-2012
Source: PLoS Negl Trop Dis. 2015 Oct 20;9(10):e0004154. doi: 10.1371/journal.pntd.0004154 (PMC4618931; doi:10.1371/journal.pntd.0004154)
Supplement: S1 File — (DOCX) [file pntd.0004154.s002.docx]

**Suplementary File**

*PAPER IN PRESS*

Supporting Information Legends: Impact of imported Hydatidosis

|  | **Native population**  **N (%)** | **Migrants population**  **N (%)** |
| --- | --- | --- |
| **Nacionality** | 534 (97.1) | 16 (2.9) |
| **Mean Age ± SD** | 61.5±20.1 | 34.6±12.8 |

A retrospective descriptive study of patients diagnosed with Cystic Echinococcosis in the Complejo Asistencial Universitario de Salamanca (CAUSA) between January 1998 and December 2014 was designed. 550 patients with new CE-related diagnosis were registered in CAUSA, of them 16 (2.9%) are inmigrants.

**Principal authors of the work**

1. **Angela Romero-Alegria,** MD. Servicio de Medicina Interna. Complejo Asistencial Universitario de Salamanca (CAUSA). Instituto de investigación Biomédica de Salamanca (IBSAL). Centro de Investigación de Enfermedades Tropicales de la Universidad de Salamanca (CIETUS), Universidad de Salamanca. Paseo San Vicente 58-182, 37007, Salamanca Spain. Tel +34 923291306.Fax +34923291131.

2. **Moncef Belhassen-García, MD, PhD**. Servicio de Medicina Interna. Sección de Enfermedades Infecciosas. Complejo Asistencial Universitario de Salamanca (CAUSA). Instituto de investigación Biomédica de Salamanca (IBSAL).Centro de Investigación de Enfermedades Tropicales de la Universidad de Salamanca (CIETUS), Universidad de Salamanca. Paseo San Vicente 58-182, 37007, Salamanca Spain. Tel +34 923291306.Fax +34923291131. Email: [mbelhassen@hotmail.com](mailto:mbelhassen@hotmail.com).
